# Enhanced attitude tracking control of spacecraft via inner and outer loop sliding mode design

**DOI:** 10.1371/journal.pone.0328622

**Published:** 2025-07-17

**Authors:** Xinxiang Fang, Ming Wen

**Affiliations:** 1 Hunan Mechanical and Electrical Ploytechnic, Changsha, Hunan, China; 2 Hunan Mingxiang Aviation Technology Co., Ltd., Changsha, Hunan, China; University of Hull, UNITED KINGDOM OF GREAT BRITAIN AND NORTHERN IRELAND

## Abstract

The significance of spacecraft in modern aerospace and technology is indisputable, with attitude control systems (ACS) playing a vital role in ensuring mission accuracy and safety. This study presents a dual-loop sliding mode variable structure control approach for spacecraft attitude regulation, leveraging integral sliding mode control (SMC) to design robust switching functions. The outer loop generates the angular velocity command $\omega_{c}$ by tracking the desired attitude angle θ, while the inner loop ensures accurate torque control based on this command. The control architecture comprises an inner velocity loop and an outer position loop, with the inner loop deliberately designed to converge faster, ensuring overall system stability. Numerical simulations validate the proposed strategy’s effectiveness and highlight its potential for real-world aerospace applications.

## 1 Introduction

Spacecraft are engineered to function beyond Earth’s atmosphere and encompass various types such as satellites [1], space shuttles [2], and probes [3]. Their primary roles involve scientific research, communications, Earth monitoring, weather observation, and exploration of deeper space. These missions expand our knowledge of the cosmos, spur technological progress, and impact economic and scientific fields significantly [4–6]. An essential focus in this field is the advancement of spacecraft attitude control, which plays a critical role in ensuring accurate positioning and stable operations required for scientific research, Earth observation, satellite communication, precise navigation, and deep space exploration. This technology draws upon various disciplines, including dynamics, control theory, algorithm development, sensor systems, and actuators [7–9]. Progress in these areas not only enhances spacecraft capabilities but also drives innovation in related high-tech domains, underscoring the importance of attitude control research in the aerospace industry and beyond.

Many scholars conduct research on spacecraft attitude control, a field that attracts attention from experts and research institutions worldwide due to its complexity and importance. S. Ullah *et al*. proposed a neuro-fuzzy-based adaptive integral super-twisting sliding mode control (SMC) method for quadcopter control. Theoretical analysis and simulations demonstrate that it outperforms traditional integral backstepping control in terms of hover stability and control performance [10]. In [11], the issue of attitude control for spacecraft equipped with gas jet or momentum exchange actuators is discussed. In [12], a localized solution for spacecraft control based on dynamic attitude feedback is introduced, and its practicality is confirmed through simulation results. In [13], Yin and Xiao, among others, explored the theoretical and practical approaches to implementing Fault Tolerant Control (FTC) in spacecraft attitude control systems (ACS), and provided an overview of the issues encountered in the design of spacecraft attitude control. In [14], the control and state estimation of Unmanned Aerial Vehicles (UAVs) face challenges due to nonlinear dynamic complexity and uncertainties such as wind disturbances and sensor noise. Zainab Akhtar proposed an altitude and attitude tracking control method for quadrotors based on an adaptive Multi-layer Neural Network (MLNN) Luenberger observer. The method employs a modified back-propagation algorithm for state estimation and integrates a sliding mode controller. Simulation results show that this approach outperforms traditional Sliding Mode Observers (SMOs) and Single Hidden Layer Neural Network (SHLNN) observers. In [15], Bustan and Hosseini and their colleagues developed an adaptive attitude controller with fault tolerance, using variable structure control to address unknown actuator faults, control input saturation, and external disturbances. They verified the system’s stability in the presence of disturbances using Lyapunov techniques and quaternion analysis. Furthermore, numerical simulations validated its capability to sustain high performance in attitude control amidst various faults and disruptions. In [16], in response to the complex disturbances and uncertainties in the space environment, Zhou and Ling and their colleagues developed a new method for constructing satellite attitude control models using system identification techniques. They successfully applied this method to the ACS of two satellites, demonstrating its practicality and effectiveness. In [17], Tsiotras and Shen, among others, proposed a new control strategy designed for the integrated power and ACS of satellites, aimed at achieving precise attitude tracking through wheel control. In [18], Hu and Xiao developed an innovative fault-tolerant attitude control method for flexible spacecraft facing actuator failures and uncertainties in inertia parameters. Through numerical simulations, they demonstrated that this control strategy not only maintains the performance of the closed-loop system but also exhibits superior fault tolerance and robustness.

In spacecraft ACS, numerous established control technologies are utilized, including Proportional Integral and Derivative (PID) control [19–22], adaptive control [23–25], robust control [26–29], and SMC methods [30–33]. These technologies enhance the performance and reliability of the ACS through various mechanisms. In [34], Noordin and Mohd, along with their colleagues, introduced an auto-tuning adaptive PID control system designed for quadrotors to address stability issues related to attitude and position in the presence of parameter uncertainties and external disturbances. Simulations have demonstrated that this control approach successfully ensures accurate tracking of both the attitude and position of the quadrotors. In [35], Zainab Akhtar proposes a novel control design method for UAV systems based on a MLNN observer, with both numerical design and practical implementation. An adaptive MLNN-based Luenberger observer is first developed for state estimation using a modified back-propagation algorithm. To evaluate the practical applicability of the proposed method, hardware-in-the-loop simulations are conducted using a Pixhawk 6X flight controller interfaced with Mission Planner software, followed by real-world experiments on an F450 quadrotor platform, demonstrating the stability and excellent tracking performance of the proposed MLNN observer-based sliding mode control scheme. In a related study, Bohlouri investigated an observer-based enhanced PID controller tailored for satellite attitude control challenges in the presence of disturbances and uncertainties. Simulation results showed that this improved PID controller achieves about 15% greater control precision than conventional methods when dealing with uncertain rotational inertia [36]. In [37], Mallikarjunan, Nesbitt, and their collaborators employed an ${ℒ}_{1}$ adaptive control approach to demonstrate robust and accurate attitude tracking performance for unmanned aerial vehicles, even in the presence of model uncertainties and environmental disturbances. Meanwhile, in [29], Hu, Li, and co-authors proposed a novel robust nonlinear control strategy aimed at ensuring the attitude stability of rigid spacecraft, explicitly considering velocity and torque constraints. They formulated a nonlinear feedback control law and validated its effectiveness in attitude regulation through a practical case study. In [38], S. Ullah and his team proposed a control method for underactuated electromechanical nonlinear systems based on backstepping sliding mode control. Both theoretical analysis and simulation results demonstrate that, compared to traditional sliding mode control, this method offers significant advantages in reducing low-frequency vibrations and enhancing system robustness. In [39], Bang and Ha proposed a new SMC strategy for the three-axis attitude maneuver of flexible spacecraft models. In [40], Yeh proposed two types of nonlinear attitude controllers for spacecraft: a sliding mode attitude tracking controller and a sliding mode adaptive attitude tracking controller. These controllers primarily adjust the orientation to achieve precise positioning of the spacecraft. [41], Yogi and Tripathi introduced an adaptive integral sliding mode control (ISMC) strategy for quadrotors to ensure rapid convergence within a finite time while reducing oscillations. Given the high uncertainty and external disturbances in quadrotor dynamics, a novel fully connected recursive neural network (FCRNN) controller was employed to simulate the equivalent control, and the system’s finite-time stability was ensured through Lyapunov stability theory. In addition, S. Ullah, Q. Khan, and others proposed a robust control method based on integral backstepping integral sliding mode control (IBISMC) to asymptotically stabilize a class of underactuated nonlinear electromechanical systems (UNEMSs) at desired equilibrium points. By applying model transformation and block-wise design combined with the integral SMC approach, the method effectively achieves zero steady-state error and strong robustness against disturbances and uncertainties, with its superiority validated through simulation results on a cart-pendulum system [42].

Building upon prior studies, this paper proposes an enhanced dual-loop SMC strategy for spacecraft attitude regulation, specifically designed to effectively counteract external disturbances. The primary innovations and contributions of this work can be summarized as follows:

1. This study proposes a dual-loop sliding mode variable structure control strategy tailored for a specific class of spacecraft. By employing integral SMC techniques to design the switching functions, the robustness and adaptability of the control system are significantly enhanced.
2. In this framework, the outer control loop is responsible for tracking the angle θ and generating the angular velocity command $\omega_{c}$, while the inner loop dynamically adjusts its control laws based on this command to ensure high tracking accuracy. This dual-loop configuration optimizes command transmission and execution, thereby improving the system’s response speed and positioning precision.
3. Furthermore, the control system is deliberately designed so that the convergence speed of the inner velocity loop exceeds that of the outer position loop. This hierarchical design ensures the overall stability of the control system and effectively mitigates potential oscillations and instabilities. Numerical simulations are conducted to validate the effectiveness of the proposed strategy, demonstrating its promising potential for practical spacecraft attitude control applications and providing robust technical support for future missions.

The structure of the paper is outlined as follows: Sect 2 introduces the system modeling and problem formulation, including key assumptions and a clear definition of the control objectives. Sect 3 details the proposed dual-loop sliding mode control approach, with a focus on the design of both outer and inner loop controllers. Sect 4 presents numerical simulation results conducted in MATLAB/Simulink to evaluate the robustness and disturbance rejection capabilities of the proposed method. Finally, Sect 5 concludes the paper by summarizing the main contributions and outlining potential directions for future research.

## 2 Model transformation and problem description

### 2.1 Dynamic model of spacecraft

**Remark 1**: Spacecraft attitude control systems are characterized by pronounced nonlinearity and strong dynamic coupling, rendering traditional linear control methods insufficient. Sliding mode variable structure control provides a robust alternative by formulating control laws capable of accommodating these nonlinear dynamics while effectively managing uncertainties and external disturbances encountered in challenging aerospace environments.

**Remark 2**: The control framework proposed in this paper integrates both fundamental physical parameters—such as the inertia matrix and angular velocity—and advanced control strategies like sliding mode variable structure control. The design also explicitly accounts for dynamic coupling and system nonlinearities, highlighting the intricate and comprehensive nature of spacecraft attitude control engineering.

First, a coordinate system is defined, where the point *o* represents the center of mass of the spacecraft. The body-fixed coordinate system is denoted as oxyz, and the inertial coordinate system, used by the spacecraft’s computer, is represented as *OXYZ*, where *O* is the origin of the inertial frame. The dynamics equation for the angular velocity of the spacecraft rotating about its center of mass in the body coordinate system can be described by the following formula:

$$J\dot{\omega }=-\Omega J\omega +M+d$$ (1)

where, $J\in {\mathbb{R}}^{3\times 3}$ represents the rotational inertia matrix of the spacecraft about its center of mass; $\omega ={\left[\begin{array}{ccc} \omega_{x} & \omega_{y} & \omega_{z} \end{array}\right]}^{T}$ denotes the angular velocity vector of the spacecraft in its own body coordinate system oxyz; $M={\left[\begin{array}{ccc} M_{x} & M_{y} & M_{z} \end{array}\right]}^{T}$ is the control torque vector defined in the same coordinate system; and $d={\left[\begin{array}{ccc} d_{x} & d_{y} & d_{z} \end{array}\right]}^{T}$ includes model uncertainties and external disturbance torques of the spacecraft.

The matrices J and Ω are defined as follows:

$$J=\left(\begin{array}{ccc} J_{xx} & -J_{xy} & -J_{xz}\\ -J_{yx} & J_{yy} & -J_{yz}\\ -J_{zx} & -J_{zy} & J_{zz} \end{array}\right)$$ (2)

$$\Omega =\left(\begin{array}{ccc} 0 & -\omega_{z} & -\omega_{y}\\ \omega_{z} & 0 & -\omega_{x}\\ -\omega_{y} & \omega_{x} & 0 \end{array}\right)$$ (3)

**Assumption 1**: It is assumed that the spacecraft’s moment of inertia matrix *J* is constant and known throughout the considered operational period, a common simplification in control design. This assumption neglects potential changes in inertia due to fuel consumption or other variable loads that might affect the system dynamics.

**Assumption 2**: It is assumed that external disturbances d and model uncertainties can be effectively mitigated through appropriate control strategies, such as SMC [43–45]. This assumption is based on the robustness of the control approach, which is expected to maintain system performance even under unknown or dynamically changing external conditions.

**Assumption 3**: It is assumed that all sensors and actuators used for measuring and executing control commands are fault-free and perform optimally, with no delays or loss of accuracy. In practical applications, however, the actual performance of these devices and potential failure modes should be considered, along with their impact on the control system’s performance.

When the spacecraft rotates around its center of mass in the sequence of pitch, yaw, and roll, the dynamics equations for the attitude angles are expressed as follows:

$$\dot{\theta }=R(\theta )\omega$$ (4)

Here, $\theta ={\left[\begin{array}{ccc} \gamma & \psi & \varphi \end{array}\right]}^{T}$ represents the attitude angles of the spacecraft, with γ as the roll angle, ψ as the yaw angle, and φ as the pitch angle.

The matrix R(θ) is determined by the following formula:

$$R(\theta )=\left(\begin{array}{ccc} 1 & \tan\psi \sin\gamma & \tan\psi \cos\gamma \\ 0 & \cos\gamma & -\sin\gamma \\ 0 & \frac{\sin\gamma }{\cos\psi } & \frac{\cos\gamma }{\cos\psi } \end{array}\right)$$ (5)

Assuming the ideal attitude angles are $\theta_{c}={\left[\begin{array}{ccc} \gamma c & \psi_{c} & \varphi_{c} \end{array}\right]}^{T}$, the dynamic equations of the spacecraft can be described as follows:

$$\left\{ \begin{array}{c} J\dot{\omega }=-\Omega J\omega +M+d\\ \dot{\theta }=R(\theta )\omega \\ y=\theta \end{array}\right.$$ (6)

Then

$$\ddot{\theta }=\dot{R}(\theta )\omega +R(\theta )\dot{\omega }\text{, then }\dot{\omega }=R^{-1}(\theta )(\ddot{\theta }-\dot{R}(\theta )\omega )$$ (7)

It can be obtained from the above formula

$$J^{-1}(\theta )(\ddot{\theta }-\dot{R}(\theta )\omega )=-\Omega J\omega +M+d$$ (8)

Then

$$\ddot{\theta }=\dot{R}(\theta )\omega +R(\theta )J^{-1}(-\Omega J\omega +M+d)$$ (9)

**Remark 3**: This model outlines the dynamics of spacecraft attitude control through a series of equations. Eqs (4) and (5) establish how attitude angles and angular velocities are interconnected, using a transformation matrix R(θ). Eq (6) integrates external influences such as torques and disturbances, highlighting the system’s nonlinear dynamics, while Eq (7) explains the effects of angular velocity changes on attitude. By designing control torques M, the system can ensure that the spacecraft’s actual attitude y closely follows the desired attitude $\theta_{c}$. This precise control is crucial for the successful orientation and navigation of spacecraft in various aerospace operations.

### 2.2 Control objectives

**Fig 1:**
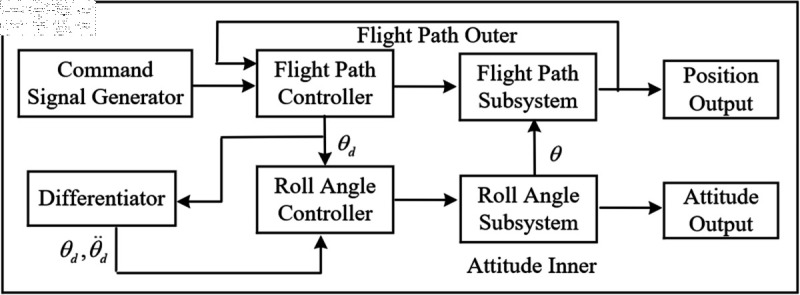
The schematic diagram of double loop control logic.

The primary objective of this paper is to design and validate a dual-loop sliding mode variable structure control strategy tailored for the attitude control of a specific class of spacecraft. The proposed approach leverages integral sliding mode control (SMC) to construct robust switching functions, enabling accurate tracking of the spacecraft’s attitude angle θ and angular velocity $\omega_{c}$ through the coordinated operation of the outer and inner control loops. In this framework, the outer loop is responsible for monitoring the attitude angle θ and generating the desired angular velocity command $\omega_{c}$, which is then transmitted to the inner loop. The inner loop subsequently adjusts its control laws to track the commanded angular velocity with high precision. This hierarchical design ensures that the inner loop responds more rapidly than the outer loop, contributing to the overall stability and responsiveness of the control system. The effectiveness and robustness of the proposed strategy are confirmed through numerical simulations, demonstrating its potential to improve both the precision and operational reliability of spacecraft attitude control in real-world missions. A logical representation of the dual-loop control architecture is provided in Fig 1.

## 3 Design of dual-loop sliding mode control law

### 3.1 Traditional sliding mode control law

Define the tracking error as $\theta_{e}=\theta_{c}-\theta$, and establish the sliding surface as:

$$s=c_{1}\theta_{e}+{\dot{\theta }}_{e}$$ (10)

Here, $c_{1}=\mathrm{diag}\left\{c_{11},c_{12},c_{13}\right\}>0$ is a positive gain matrix. The rate of change of the sliding surface is expressed as:

$$\dot{s}=c_{1}{\dot{\theta }}_{e}+{\ddot{\theta }}_{e}=c_{1}{\dot{\theta }}_{e}+{\ddot{\theta }}_{c}-\left(\dot{R}(\theta )\Omega +R(\theta )J^{-1}(-\theta J\omega +M+d)\right)$$ (11)

To counter disturbances, the following control law is designed:

$$M=\Omega J\omega +JR^{-1}(\theta )\left(D\mathrm{sgn}(s)+c_{1}{\dot{\theta }}_{c}+{\ddot{\theta }}_{c}-\dot{R}(\theta )\Omega \right)$$ (12)

Substituting this into the expression for $\dot{s}$, we obtain:

$$\dot{s}=-D\mathrm{sgn}(s)-R(\theta )J^{-1}d$$ (13)

Assuming the disturbance boundary satisfies $\left|R(\theta )J^{-1}d\right|⩽D$, we have:

$$s^{T}\dot{s}=-\left\|s^{T}\right\|D-s^{T}R(\theta )J^{-1}d⩽0$$ (14)

To further enhance the system’s resistance to disturbances, an integral sliding mode control strategy based on a dual-loop sliding mode is proposed. The inner loop control is utilized to eliminate the effects of disturbances, while the outer loop ensures precise attitude tracking, thereby enhancing the overall system stability and efficiency.

**Remark 4**: The traditional SMC law delineated here utilizes a robust approach for tracking control, tailored to handle uncertainties and disturbances effectively within dynamic systems. The defined sliding surface (10) and its derivative (11) form the foundation for the control strategy, ensuring rapid convergence and error correction. The control law (12) specifically includes a discontinuous term that counteracts disturbances and guarantees the sliding condition (13), leading to the system’s stability as verified by the inequality (14). This robustness is particularly vital in applications where precision and reliability are crucial, such as in aerospace and robotics.

### 3.2 Outer loop sliding mode control

The external loop sliding mode control primarily focuses on designing the angular velocity command $\omega_{c}$. It uses $\omega_{e}=\omega_{c}\;-\;\omega$ as the virtual control input for the external loop system to achieve precise tracking of the attitude angle. This approach enables the control system to respond more effectively to attitude adjustments, thereby enhancing the overall system stability and response speed.

The design of the external sliding surface is as follows:

$$s_{w}=\theta_{e}+K_{l}\int_{0}^{t}\theta_{e}\;dt,\;s_{w}\in R^{3}$$ (15)

where, $K_{1}=\mathrm{diag}\left\{k_{11},k_{12},k_{13}\right\}>0$ is a positive gain matrix. By appropriately selecting the gain matrix, the system’s tracking error can be gradually stabilized on the desired sliding surface, the atual control output M cannot be directly obtained due to the absence of $\ddot{\theta }$. This issue can be addressed by designing the control function for the inner loop.

The angular velocity command $\omega_{c}$ is set as a virtual control input for the angular velocity ω. Define the error $\omega_{e}=\omega_{c}-\omega$. Based on the dynamics relationship:

$$\dot{\theta }=R(\theta )\left(\omega_{c}-\left(\omega_{c}-\omega \right)\right)=R(\theta )\omega_{c}-R(\theta )\omega$$ (16)

The inner loop control aims to eliminate $\omega_{e}$, i.e., the discrepancy between the angular velocity command and the actual angular velocity, thus ensuring higher precision in attitude tracking.

According to Eq (15), the dynamics of the external loop SMC can be derived as follows:

$${\dot{s}}_{w}={\dot{\theta }}_{c}+K_{1}\theta_{c}={\dot{\theta }}_{c}-\dot{\theta }+K_{1}\theta_{c}={\dot{\theta }}_{c}-R(\theta )\omega_{c}+R(\theta )\omega_{c}+K_{1}\theta_{c}$$ (17)

Furthermore, due to the requirements of the inner loop sliding mode control, $\omega_{c}$ must be a differentiable function. Thus, the virtual control input for the external loop can be designed as:

$$\omega_{c}=R^{-1}(\theta )\left({\dot{\theta }}_{c}+K_{1}\theta_{e}\right)+R^{-1}(\theta )\rho_{1}s_{w}$$ (18)

Here, $\rho_{1}>0$ is a positive control gain, used to enhance the system’s resistance to disturbances and improve stability.

Considering the following Lyapunov function:

$$V_{1}=\frac{1}{2}s_{w}^{T}s_{w}$$ (19)

The time derivative of $V_{1}$ can be expressed as:

$${\dot{V}}_{1}=s_{w}^{T}{\dot{s}}_{w}=s_{w}^{T}\left({\dot{\theta }}_{c}-R(\theta )\omega_{c}+R(\theta )\omega_{c}+K_{1}\theta_{c}\right)\;=s_{w}^{T}\left\{{\dot{\theta }}_{c}-R(\theta )\left[R^{-1}(\theta )\left({\dot{\theta }}_{c}+K_{1}\theta_{e}\right)+R^{-1}(\theta )\rho_{1}s_{w}\right]+R(\theta )\omega_{c}+K_{1}\theta_{e}\right\}\;=-\rho_{1}{\left\|s_{w}\right\|}^{2}+s_{w}^{T}R(\theta )\omega_{c}$$ (20)

It is apparent from the equation that to ensure ${\dot{V}}_{1}\leq 0$, it is crucial to minimize the error $\omega_{e}$. This requirement underscores the importance of designing a rapid-convergence inner-loop SMC.

### 3.3 Inner loop sliding mode control

As illustrated, to synchronize the target angular velocity $\omega_{c}$ with ω, an inner-loop sliding mode control strategy has been developed. The essence of this control approach lies in the use of control law M to ensure the convergence of $\omega_{c}-\omega$ to zero. Additionally, an integral SMC method has been employed to construct the sliding mode function, expressed as:

$$s_{n}=\omega_{e}+K_{2}\int_{0}^{t}\omega_{e}dt$$ (21)

where, $K_{2}=\mathrm{diag}k_{21},k_{22},k_{23}$ represents a diagonal matrix of gains, which regulates the response speed and stability of the control.

Derived from Eqs (6) and (21), we obtain:

$${\dot{s}}_{n}={\dot{\omega }}_{e}+K_{2}\omega_{e}={\dot{\omega }}_{c}+J^{-1}(\Omega J\omega -M-d)+K_{2}\omega_{e}$$ (22)

To achieve effective control, the inner-loop control law is designed as follows:

$$M=\Omega J\omega +J\left({\dot{\omega }}_{c}+K_{2}\omega_{e}\right)+\rho_{2}\mathrm{sign}\left(s_{n}\right)+ks_{n}$$ (23)

where, $\rho_{2}>\max\left|d_{i}\right|,k=\left[\begin{array}{cc} k & 0\\ 0 & k \end{array}\right]>0$.

To analyze system stability, consider the following Lyapunov function:

$$V_{2}=\frac{1}{2}s_{n}^{T}Js_{n}$$ (24)

Then

$${\dot{V}}_{2}=s_{n}^{T}J{\dot{s}}_{n}=-\rho_{2}\sum_{i=1}^{3}\left|s_{n}\right|-s_{n}^{T}d-k{\left\|s_{n}\right\|}^{2}⩽-k{\left\|s_{n}\right\|}^{2}$$ (25)

Assuming $J_{\text{max}}=\max J$ and setting $k⩾\frac{1}{2}J_{\text{max}}$, we ensure that $k|sn{|}^{2}⩾\frac{1}{2}sn^{T}Jsn$. Consequently, $-k|sn|⩽-\frac{1}{2}sn^{T}Jsn=-V_{2}$, leading to ${\dot{V}}_{2}⩽-V_{2}$. This implies that $V_{2}$ will exponentially decay at the rate of $e^{-t}$, approaching zero as t→∞.

**Remark 5**: In the design of inner and outer loop control systems, the dynamic performance of the inner loop must surpass that of the outer loop to ensure the stability of the entire control system. A common engineering practice is to design the inner loop with a faster convergence rate than the outer loop. In this algorithm, we set $K_{2}⩾K_{1}$ and choose sufficiently large values of k to quickly eliminate the angular velocity error $\omega_{e}$, ensuring ${\dot{V}}_{1}⩽0$ and maintaining a faster convergence rate in the inner loop compared to the outer loop.

**Remark 6**: The selection of gain matrices $K_{1}$, $K_{2}$, $c_{1}$, and control coefficients $\rho_{1}$, $\rho_{2}$, and k is based on ensuring system stability through Lyapunov analysis and achieving a faster convergence in the inner loop. The inner loop gains are chosen to satisfy $K_{2}\geq K_{1}$ and $k\geq \frac{1}{2}J\max$, ensuring exponential convergence. All selected gain values are listed in Table 1 for clarity.

**Table 1:** Sliding mode control gains.

Parameter	Description	Value
$c_{1}$	Gain matrix for traditional SMC surface	diag{3,3,3}
$K_{1}$	Outer loop integral gain matrix	diag{2,2,2}
$K_{2}$	Inner loop integral gain matrix	diag{5,5,5}
$\rho_{1}$	Control gain for outer loop robustness	3
$\rho_{2}$	Control gain for inner loop robustness	5
k	Damping gain matrix	diag{10,10,10}

## 4 Numerical simulation

The numerical simulation verification in this paper is based on MATLAB/SIMULINK. To further validate the effectiveness of the proposed control method, we compare it with the PD control method [46]. In addition, the main simulation program of this paper can be found in supporting information (S1.pdf).

Based on model Eq (6), consider a spacecraft with inertia parameters $J_{xx}=78,J_{yy}=80,J_{xz}=99$, and $J_{xy}=J_{yz}=J_{xx}=0$, without any modeling uncertainties. The disturbance torques are modeled by the trigonometric functions $d={\left[\sin(t)\;+\;0.010{\mathrm{.88}}^{*}\cos(t)\;+\;0.05\sin(2.5*t)\;+\;0.08\right]}^{\prime }$. The spacecraft’s initial angles $\theta ={\left[\begin{array}{ccc} \gamma & \psi & \varphi \end{array}\right]}^{T}$ and initial angular velocities $\omega ={\left[\begin{array}{ccc} \omega_{x} & \omega_{y} & \omega_{\epsilon } \end{array}\right]}^{T}$ are both set to zero, with the angle tracking command given by the cosine signals $\theta_{c}={\left[\begin{array}{ccc} \cos t & \cos t & \cos t \end{array}\right]}^{T}$, and inertia matrix


$$J=\left(\begin{array}{ccc} 78 & 0 & 0\\ 0 & 850 & 0\\ 0 & 0 & 99 \end{array}\right)$$

**Fig 2:**
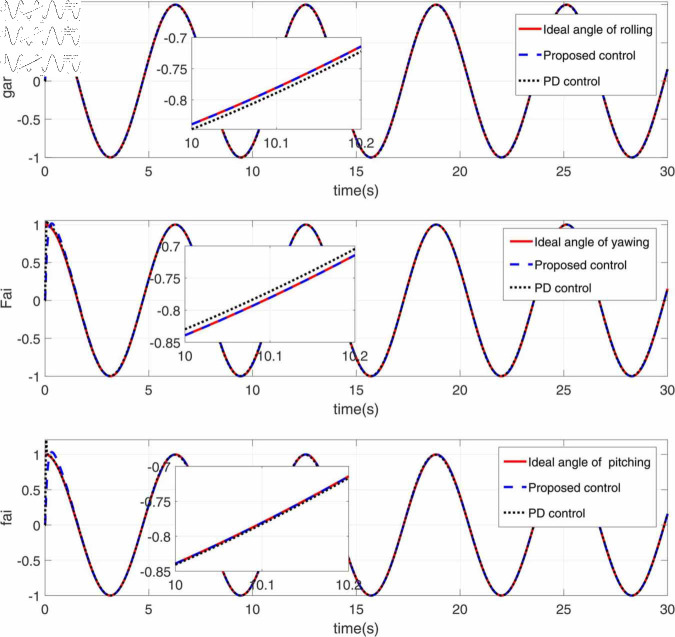
Tracking response diagram of roll angle, yaw angle and pitch angle.

The designed virtual outer loop and inner loop sliding mode control laws for this system are as follows: The outer loop control parameters are set to $\rho_{1}=5$ with a gain matrix $K_{1}=\left(\begin{array}{ccc} 0.35 & 0 & 0\\ 0 & 0.35 & 0\\ 0 & 0 & 0.35 \end{array}\right)$. The parameters for the inner loop sliding mode control law are $K_{2}=\left(\begin{array}{ccc} 8 & 0 & 0\\ 0 & 8 & 0\\ 0 & 0 & 8 \end{array}\right)$, $k=\left(\begin{array}{ccc} 80 & 0 & 0\\ 0 & 80 & 0\\ 0 & 0 & 80 \end{array}\right)$. In the inner loop control, the switching function is replaced by a saturation function, with saturation parameters set to $\Delta_{i}=0.015$, where i=1,2,3. The simulation results are depicted in Figs 2 and 4.

**Fig 3:**
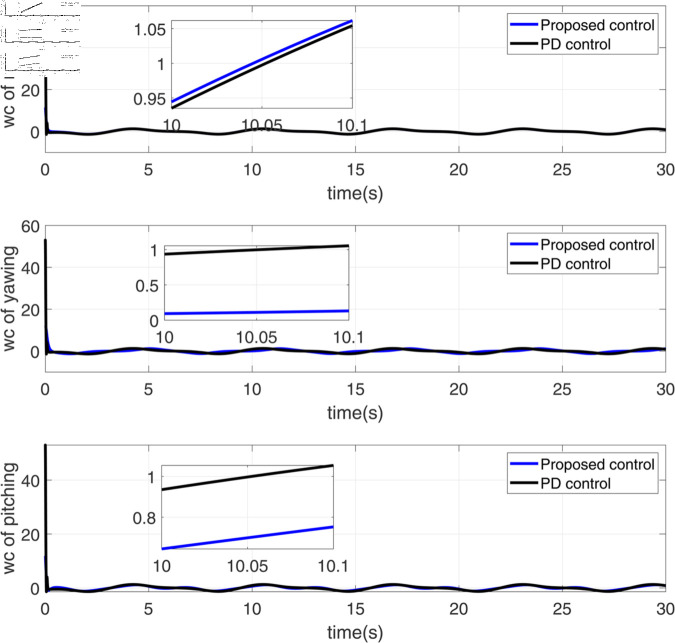
Response diagram of rolling moment, deflection moment and pitching moment.

**Fig 4:**
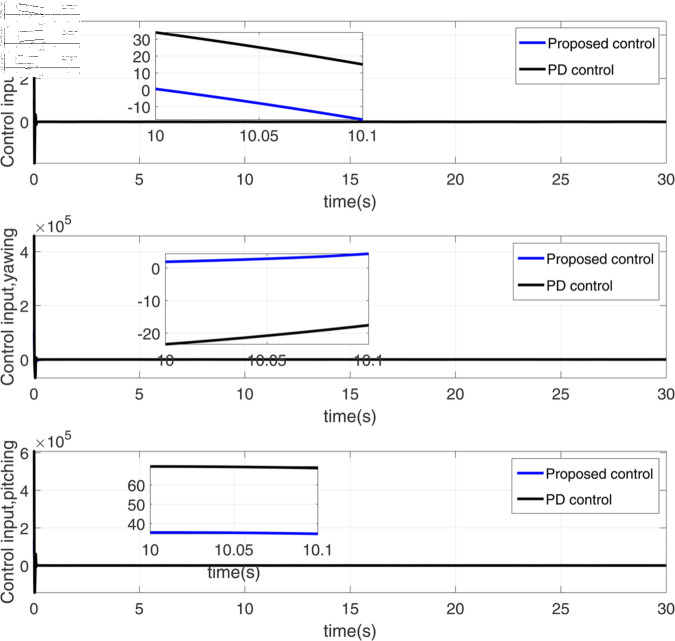
Response diagram of three attitude angle control inputs.

Fig 2 presents the tracking response of the roll, yaw, and pitch angles over time. The red line represents the ideal angle for each motion (roll, yaw, and pitch), the blue line represents the tracking performance of the proposed control method, the black dashed line shows the tracking performance of the PD control method. The insets zoom in on specific regions around *t* = 10 seconds, allowing for a closer comparison between the proposed and PD control methods. Overall, the proposed control method performs better than the PD control method, with smaller deviations from the ideal angles.

Fig 3 presents the diagram of rolling moment, deflection moment, and pitching moment. This figure focuses on the control inputs related to the rolling, yawing, and pitching moments. The responses of the proposed control and PD control are plotted over time. The control inputs for all three moments (rolling, yawing, and pitching) are plotted on the same scale, with the proposed control method generally maintaining smaller fluctuations compared to the PD control. In all three cases, the proposed control method provides smoother responses with fewer large deviations, indicating better control precision. The insets again zoom in on smaller time intervals, providing a clearer comparison at key moments of the control input.

Fig 4 illustrates the time-domain response of three control inputs governing aircraft or spacecraft attitude angles (typically roll, pitch, and yaw). The diagram plots control effort (e.g., actuator commands, torque demands, or surface deflections) on the vertical axis against time on the horizontal axis. Each curve represents one attitude control channel, showing how the control system modulates inputs (such as thruster pulses or control surface movements) to achieve desired angular orientations. Key features include transient phases (initial adjustments), stabilization periods (convergence toward setpoints), and steady-state behavior (maintenance of target angles), with variations in amplitude and timing reflecting dynamic system requirements and controller tuning. Overshoot, oscillations, or settling times may indicate performance characteristics or design trade-offs.

## 5 Conclusion

This study successfully implements a dual-loop sliding mode variable structure control strategy for precise attitude control of a specific spacecraft, significantly enhancing the accuracy and safety of mission execution. By coordinating the internal and external loops, the system optimizes command transmission and execution, resulting in improved response speed and control precision.

However, certain limitations may arise when applying the proposed control strategy to highly complex dynamic environments, such as sensitivity to unmodeled dynamics, actuator saturation, and the impact of severe external disturbances. To address these challenges, future research will focus on the following directions:

Optimization of Control Parameters: Developing adaptive control techniques to dynamically adjust the control parameters in real-time, thereby improving the robustness of the system against unmodeled dynamics and actuator limitations.

Disturbance Rejection and Fault Tolerance: Investigating advanced disturbance rejection methods, such as robust disturbance observer-based strategies, to mitigate the effect of external disturbances and ensure reliable performance in harsh conditions.

Comprehensive Experimental Validation: Conducting extensive experiments under a wider range of real-world conditions to further validate the practical feasibility and effectiveness of the proposed control strategy in both nominal and extreme scenarios.

Extension to Other Platforms: Extending the proposed control framework to other dynamic systems, such as unmanned aerial vehicles (UAVs), drones, and robotic platforms, to explore its broader application scope and adaptability to various dynamic environments.

## Supporting information

S1 File Paper program (PDF)
